# Controversies about Interspinous Process Devices in the Treatment of Degenerative Lumbar Spine Diseases: Past, Present, and Future

**DOI:** 10.1155/2014/975052

**Published:** 2014-04-13

**Authors:** Roberto Gazzeri, Marcelo Galarza, Alex Alfieri

**Affiliations:** ^1^Department of Neurosurgery, San Giovanni-Addolorata Hospital, Via Amba Aradam 9, 00184 Rome, Italy; ^2^Regional Service of Neurosurgery, “Virgen de la Arrixaca” University Hospital, Avenida Primero Mayo, El Palmar, 30120 Murcia, Spain; ^3^Klinik für Neurochirurgie und Wirbelsäulenchirurgie, Fehrbelliner Straße 38, 16816 Neuruppin, Germany

## Abstract

A large number of interspinous process devices (IPD) have been recently introduced to the lumbar spine market as an alternative to conventional decompressive surgery in managing symptomatic lumbar spinal pathology, especially in the older population. Despite the fact that they are composed of a wide range of different materials including titanium, polyetheretherketone, and elastomeric compounds, the aim of these devices is to unload spine, restoring foraminal height, and stabilize the spine by distracting the spinous processes. Although the initial reports represented the IPD as a safe, effective, and minimally invasive surgical alternative for relief of neurological symptoms in patients with low back degenerative diseases, recent studies have demonstrated less impressive clinical results and higher rate of failure than initially reported. The purpose of this paper is to provide a comprehensive overview on interspinous implants, their mechanisms of action, safety, cost, and effectiveness in the treatment of lumbar stenosis and degenerative disc diseases.

## 1. Introduction

The degenerative lumbar spine is associated with significant structural failure of the intervertebral disc, of the ligaments, and/or of the bone structures [1]. The typical findings are radial fissures, prolapses, endplate damage, annular protrusion, internal disc disruption, disc space narrowing, hypertrophic ligaments, hypertrophic facet joints, and osteophytes [1–4]. These degenerative changes may cause instability in advanced stages of the disease [5–19]. The clinical endpoint of these degenerations is the compression of neural structures at the level of the neural foramina or of the spinal canal.

Typically, patients complain about low back pain with or without pseudoradicular pain or dysesthesia. The actual operative “gold standard” to treat degenerative lumbar spinal disease is generally decompression with or without fusion of the affected segment [20–27]. However, some investigators began to explore novel minimally invasive approaches to stabilize the lumbar spine.

Although a growing number of different minimal invasive treatments have been introduced for the degenerative lumbar spine disease, the interspinous process devices are becoming an acceptable alternative for lumbar decompressive surgery [28–32]. However, interspinous devices are presented also as a viable option for treating a vast number of lumbar pathologies ranging from facet syndrome and discogenic low back pain to degenerative spinal stenosis, discopathy, and lumbar instability. The arising consequence is the need to understand the pathological and mechanical causes of each degenerative problem and determine the right treatment paradigm through a critical analysis of all available experimental and clinical biomechanical information [33–38]. Various authors suggest that advantages of IPD compared with standard surgical decompression techniques are the option of local anesthesia, preservation of bone and soft tissue, reduced risk of epidural scarring and cerebrospinal fluid leakage, with a shorter hospital stay and rehabilitation period, and reversibility of the surgical procedure that does not limit future surgical treatment options [39–46]. Currently, there are no long-term clinical trials for IPD: published clinical data are sparse and in the majority of cases consist of small, nonrandomized studies with short-term follow-up. In this paper we provide an overview of the current notions of the biomechanical principles of the interspinous process devices, as well as in experimental and clinical studies. These considerations are applicable with different types of interspinous spinous devices with only few differences between the distinct categories.

## 2. Pathophysiology and Mechanism of Action

The pathoanatomic feature of neurogenic intermittent claudication in lumbar degenerative diseases is the venous stasis in lumbar spine extension, causing neurologic symptoms as motor weakness in the lower extremities, pain, tingling, and sensory deficit, which make walking for a long distance impossible. The first recommended indication for the implantation of an IPD was mild and moderate intermittent neurogenic claudication from spinal stenosis [36]. The key selection criteria were (and are) that patients symptoms must be relieved by flexion of the lumbar spine. This phenomenon is called “shopping cart sign” due to the improvement in walking endurance in stenotic patients leaning forward on a shopping cart. Flexion of the stenotic lumbar spine stretches the redundant ligamentum flavum and enlarges the neural foramina, thus relieving lower extremity symptoms. Recently, most of the devices have been marketed as treatments for discogenic low back pain: posterior elements distraction unloads posterior annulus modulating the mechanical stimuli to the nociceptive nerve endings of the sinuvertebral nerve.

### 2.1. Enlargement of the Spinal Canal Area

A decisive index for the relief of the clinical signs and symptoms is the enlargement spinal canal area. The mean expansion of the spinal canal after insertion of the interspinous process devices is reported between 18% [37] and 22% [35], with significant differences between the standing, the seated neutral, and the seated extended position [45] being, respectively, 8.3%, 8.6%, and 7.9%. Cross-sectional area of the dural sac studied with a dynamic magnetic resonance imaging was reported to increase from 78 mm^2^ preoperatively to 93 mm^2^ postoperatively in the standing position, from 93 to 108 mm^2^ in the seated neutral position, and from 85 to 107 mm^2^ in the seated extended position. In a magnetic resonance imaging cadaver study, Richards et al. reported that the X-Stop increases the spinal canal area by 18% during extension [37] (Table 2).

### 2.2. Increase of the Neural Foramina Area

Neural foramina area is increased after insertion of an interspinous device. In a dynamic magnetic resonance imaging study, neural foramina were increased between 23 and 26 mm^2^ in the extended position. Another study shows that the foraminal area is increased by 25% [37] after insertion of interspinous process devices, but the foraminal width can increase up to 40%.

Richards showed in a radiological cadaver study that IPD (in their case X-Stop) increased the neural foramen area by 26% with a subarticular diameter that was increased by 50% in extension [37].

Lee et al. reported an increase of the foraminal area of 22 mm^2^ (37%) after X-Stop implantation [35].

### 2.3. Unload of the Posterior Annulus and Intradiscal Pressure

The interrelation between unloading of the discal structures and distraction of posterior lumbar elements is a much debated issue. The rabbit models suggest that prolonged disc distraction might reverse some aspects of the compression-induced degeneration with better results at the L3/L4 level [47–51]. Nevertheless, the biomechanical mechanism is not clear, because both compression and distraction cause a significant decrease in nucleus pressure; however, the compression results in a greater pressure decrease than distraction. It was theorized that reduction in pressure with distraction results from a void between both endplates [44]. The measurement of the height of the posterior disc as indirect sign of the intradiscal pressure showed an average from 0.09 to maximal 1.75 mm [11, 35, 40]. In a cadaveric disc pressure study, Swanson et al. reported that the pressures in the posterior annulus and nucleus pulposus were reduced by 63% and 41%, respectively, during extension and by 38% and 20%, respectively, in the neutral, standing position [52]. The exhaustive mechanism of the intradiscal pressure interaction with the neural structures is today not clarified. Axial loading MRI examination is seldom used to show the dynamical modification of a degenerative disc in the lumbar spine. It is performed with the patient supine and gravity is typically simulated using a compressive system comprised of a vest worn by the patient over the shoulders and upper chest attached to a footplate against which the patient's feet are braced. The platform pushes under computer control, maintaining a stable push during the examination: the load applied is 65% of the patient's weight. The examination is performed in a neutral position and after loading with axial and sagittal T2-weighted scans. The images are subsequently evaluated to identify load-induced changes. A dynamically degenerative modification of the lumbar spine has been observed when performing MRI under axial loading. Previously studies reported that disc bulging increases under loading conditions with consequent restrictions of the spinal canal area, irregular slipping, and abnormal movements of the articular facet joints, as well as increases in local scoliosis with asymmetric restrictions of the neuroforamen area [53].

### 2.4. Distraction of Interspinous Distance

Another indirect measure published is the distance between the spinous processes. The reported data [38, 40] show the persistence of the distraction over a period of two years and four years. Nevertheless, major criticism against this index is the absence of a direct correlation between the interspinous distance and the clinical symptoms. Consequently, the interspinous distance should be used only as auxiliary indicator.

### 2.5. Strength of the Spinous Processes

The lateral force required to fracture a human lumbar spinous process with varying bone densities ranges between 95–786 N with a load of average 317 N [41]. The distraction force necessary to break the lumbar spinous process ranged between 242–1 and 300 N with an average load of 339 N [54].

The lateral experimentally measured force to implant an interspinous device ranges from 11 to 150 N [41]. Based on these data, a severely osteoporotic patient may be contraindicated for interspinous device, because a fracture of the spinous process might occur intraoperatively or postoperatively.

Surgeons should be aware that the insertion of an interspinous device requires personalized forces and caution, but osteopenia is not an absolute contraindication for the operation.

### 2.6. Combining IPD Insertion and Microdiscectomy/Foraminal Decompression/Interbody Fusion

Recently various studies have been published combining the insertion of an interspinous device and microdiscectomy/foraminotomy and interbody fusion. Fuchs was the first to suggest that interspinous device can be implanted with unilateral medial or total facetectomy to stabilize the spine; however, there is no biomechanical paper to show the level of stability provided by IPD after unilateral facetectomy specially that biomechanical studies have documented the destabilizing effects of unilateral facetectomy [55, 56].

Ploumis et al. evaluated the combination of direct unilateral decompression and indirect decompression with an X-Stop in twenty-two lumbar spinal stenosis and described an effective clinical improvement at two-year follow-up [57].

Gonzalez-Blohm et al. evaluated the biomechanical performance of an interspinous fusion device as a stand-alone device, after lumbar decompression surgery, and as supplemental fixation in a posterior lumbar interbody fusion (PLIF) construct. They suggested that IPD may be a suitable device to provide immediate flexion-extension balance after a unilateral laminotomy. PLIF constructs with IPD and pedicle screws performed equivalently in flexion-extension and axial rotation, but the PLIF-bilateral pedicle screws construct was more resistant to lateral bending motions. The authors requested further biomechanical and clinical evidence to strongly support the recommendation of a stand-alone interspinous fusion device or as supplemental fixation to expandable posterior interbody cages [58].

## 3. Historical Background of Interspinous Process Devices 

The first interspinous implant for the lumbar spine was developed in the 1950s by Knowles. Owing to flaws in design, material, surgical technique, and applied indications, its use was abandoned. The first modern interspinous device, the Wallis system, was developed by Abbot Spine in 1986 and it was used primarily in patients with recurrent disc herniation [14]. It was a “floating system” that was comprised of a titanium spacer placed between the spinous processes and secured with two Dacron ligaments wrapped around the spinous processes. This system was not initially marketed commercially while waiting for long-term follow-up results. In a reported prospective trial, the application of the first generation Wallis device improved outcome in patients who underwent a second discectomy. Despite favorable results, Senegas thought that the device could be improved. A second generation of the Wallis device, slightly different in shape, and composed of polyetheretherketone (PEEK), was used with other surgical procedures, to reduce pain severity in cases of moderate disc degeneration, central spinal stenosis, and significant lower back pain. The Minns device was the first “soft” interspinous spacer indicated for sagittal plane instability [32]. The implant was fashioned out of silicone into the shape of a dumbbell to off-load the facet joints and decrease the intradiscal pressure. But despite the promising in vitro results, no further clinical application was published to date and it is unclear whether the implant advanced much further than the laboratory settings. In the 1990s, several other IDP devices displaying significant differences in design, materials, surgical techniques, and indications appeared in Europe and South America, for which there are ongoing trials of evaluation for a host of clinical indications. Kaech et al. first reported on the interspinous “U” (Coflex) suggesting that it was indicated for protection against adjacent level disc disease and restabilization of a lumbar laminectomy [59]. Caserta et al. reported on the DIAM implant, which was indicated for a number of conditions, including degenerative disc disease, herniated nucleus pulposus, and lumbar instability [29]. The X-Stop device (Medtronic, Tolochenaz, Switzerland) was approved by the US Food and Drug Administration in 2005 for the treatment of neurogenic intermittent claudication secondary to lumbar stenosis [38].

## 4. Type of Implants

Contemporary models of fusion interspinous devices have evolved from spinous process wiring with bone blocks and early device designs as the Wilson plate: the newer devices range from paired plates with teeth to U-shaped devices with wings that are attached to the spinous processes. They are intended to be an alternative to pedicle screw and rod constructs and also to aid in the stabilization of the spine with interbody fusion. Recently with greater focus on motion-preservation alternatives, interest in nonfusion interspinous devices has emerged. Interspinous fixation devices are placed under direct visualization or percutaneously using a C-arm and they can be categorized by design as static, dynamic, or fusion devices. Despite the fact that they are composed of a wide range of different materials including titanium, polyetheretherketone, bone allograft, and elastomeric compounds, the intention of the implant is to maintain a constant degree of distraction between the spinous processes. In the latest years, the spine market saw few of the world's leading medtech companies abandon their position in the hotly contested sector, where they were, at best, a bit player. Deciding that they did not want to invest any additional resources in attempting to grow its small share of the market, Abbott Spine agreed to be acquired by Zimmer Holdings Inc. and St. Francis Medical Technologies was acquired by Medtronic. We listed the most important devices that are still on the market (Table 1).

### 4.1. Static and Dynamic Interspinous Devices

**Figure 1:**
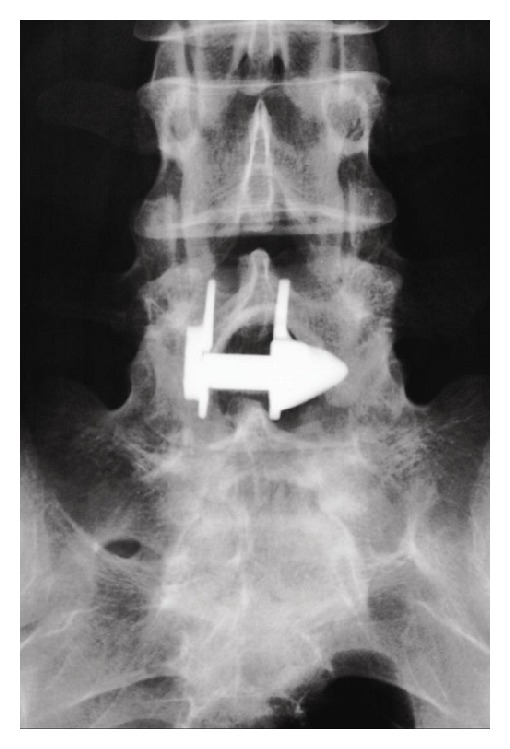
Anterior-posterior X-ray image showing an X-Stop device implanted at L4-L5 interspinous level.

**Figure 2:**
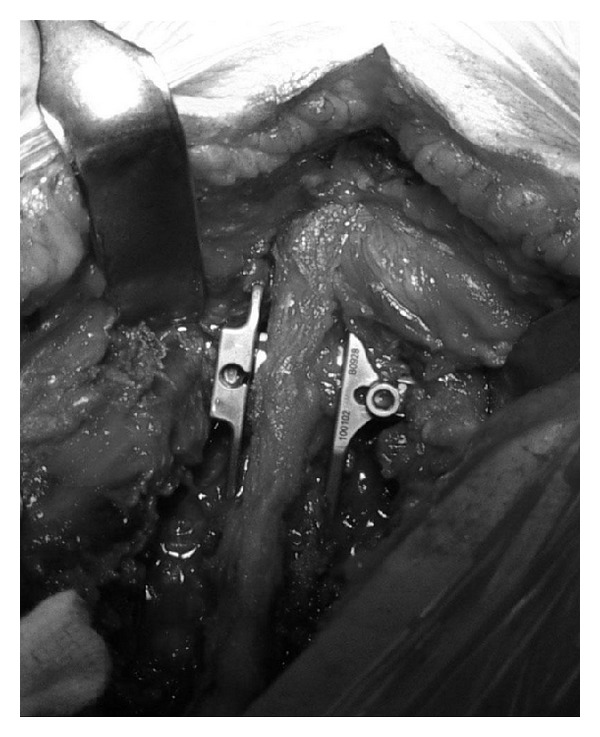
Intraoperative image shows the X-Stop interspinous device implanted cranially to a posterior fixation with pedicle screws, to avoid the topping off phenomenon.

*X-Stop (Medtronic, Tolochenaz, Switzerland, Formerly St. Francis Medical Technologies, Alameda, CA).* The X-Stop interspinous process decompression system is an interspinous spacer developed to treat patients with neurogenic intermittent claudication. It is an all-titanium (peek surrounded since end of 2004) device composed by an oval spacer, one fixed wing, one adjustable wing, and one tissue expander (Figures 1 and 2). X-Stop is the only IDP device with class I data and a prospective randomized control trial supporting its safety and efficacy compared to the nonoperative treatment. It is indicated for treatment of patients aged 50 or older suffering from pain or cramping in the legs (neurogenic intermittent claudication) secondary to a confirmed diagnosis of lumbar spinal stenosis. The X-Stop is indicated for those patients with moderately impaired physical function who experienced relief in flexion from their symptoms of leg/buttock/groin pain, with or without back pain and have undergone a regimen of at least 6 months of nonoperative treatment. The X-Stop may be implanted at one or two lumbar levels. The U.S. Food and Drug Administration approval of X-Stop interspinous decompression system was based on laboratory, mechanical, and cadaver studies and also a multicenter, prospective randomized controlled clinical study [38].

Surgical technique used was as follows: patients are placed on an operative table in a prone or right lateral decubitus position. The intervertebral level to be treated is identified by fluoroscopy. Because the implant was designed to be placed without removing any bony or soft tissues, the technique may be performed under local anesthesia. A midsagittal incision of approximately 4 cm is made over the spinous processes of the stenotic levels. The fascia is split longitudinally 2 cm to the right and to the left of the midline. It is of paramount importance to keep the supraspinous ligament intact. Paraspinal muscles are elevated off the spinous processes and medial lamina bilaterally using electrocautery. Occasionally, hypertrophied facets that block entry into the anterior interspinous space are trimmed partially to enable anterior placement of the implant. A small curve dilator is inserted across the interspinous ligament; after the correct level is verified by fluoroscopy, the small dilator is removed and the larger curve dilator is inserted. After removing the latter dilator, the sizing distractor is inserted and the interspinous space is distracted until the supraspinous ligament becomes taught. The correct implant size is indicated on the sizing instrument and the appropriately sized X-Stop implant is inserted between the spinous process.

The oval spacer separates the spinous processes and limits extension at the implanted level. The oval spacer distributes the load along the concave shape of the spinous processes. The screw hole for the universal wing on the left side is visualized and the screw is engaged. The two wings are approximated towards the midline and the screw is secured. The two lateral wings prevent migration anteriorly or laterally, and the supraspinous ligament prevents the implant from migrating posteriorly.

**Figure 3:**
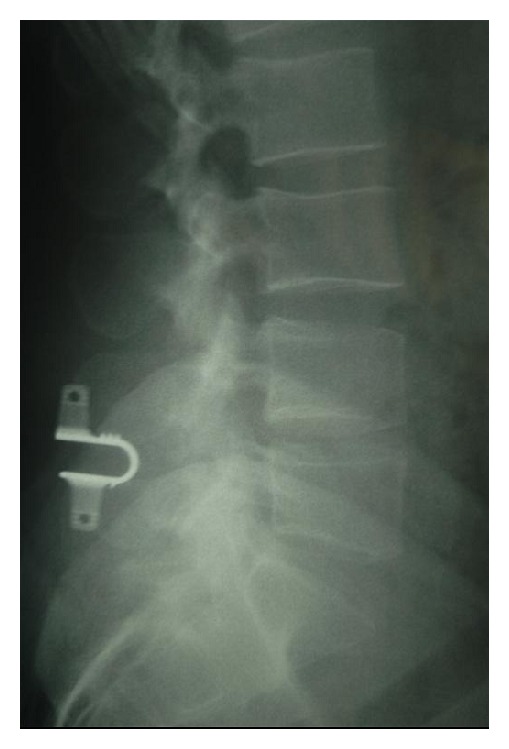
Lateral X-ray image of a Coflex implant.

*Coflex (Paradigm Spine, LLC, New York).* This device was originally developed in France by Dr. Jacques Samani in 1994, also called “interspinous U.” It is designed to be placed between two adjacent processes. It is a titanium device with a U-shaped body and two wings on each side (Figure 3). This implant is designed to permit flexion of the spine, thus restricting mobility in extension and rotation. The Coflex is FDA approved as an adjunct to fusion but is not approved as a stand-alone spacer. Although it was initially developed as a motion-preserving alternative used to treat various lumbar degenerative disorders, long-term studies from Europe suggested that the subset of patients with spinal stenosis and Grade I spondylolisthesis experienced the most significant improvement.

Surgical technique used was as follows: the patient is placed in prone position with slightly lumbar flexion. After a midline skin incision of 4–6 cm, the paraspinal muscles are stripped off the laminae. The interspinous ligament is removed and its bony attachments are resected. To define the appropriate implant size, trials are utilized. Some bony resection of the spinous process may be needed. The interspinous implant (8, 10, 12, 14, or 16 mm) is introduced tightly with gentle hammering using a mallet.

Thereafter, the wing clamps of the interspinous U are tightened against both edges of the upper and lower spinal process. In the first generation of the device, the wing clamps could be attached to the spinous processes by a suture passed through the central hole. Fixation at the spinous processes with the new generation of Coflex is possible with crimping of the wings. Coflex F has a secure anchorage to the spinous processes through rivet fixation. The depth of insertion of the Coflex can be verified under lateral fluoroscopy. The proper depth is determined if a nerve hook can be passed freely leaving 3 to 4 mm between the bottom of the Coflex and the thecal sac. 

*DIAM (Medtronic/Sofamor Danek).* The DIAM (Device for Intervertebral Assisted Motion) is an “H” shaped silicone bumper wrapped into a polyester sheath connected to artificial ligaments of the same material that is designed to support dynamically the vertebrae, restoring posterior column height and maintaining distraction of the foramina; the device acts as shock absorber, relieving stresses on both anterior and posterior elements of the spine. This device was clinically used for multiple pathologies including degenerative disc disease, canal and/or foraminal stenosis, disc herniation, black disc and facet syndrome, and topping-off.

Surgical technique used was as follows: with the patient placed in a prone position, a simple midline approach with dissection of muscles from the spinous process is performed. After opening the interspinous ligament, the DIAM device is positioned on the open inserter. The wings of the device are folded as the inserter flanges are compressed, thus the DIAM is driven as far anterior as possible using the impactor. Finally, the device is secured to the adjacent spinous processes by means of the implant's tethers. Longitudinal tension is applied and a crimper is used to secure the rivets and the excess length of the bands is cut. 

**Figure 4:**
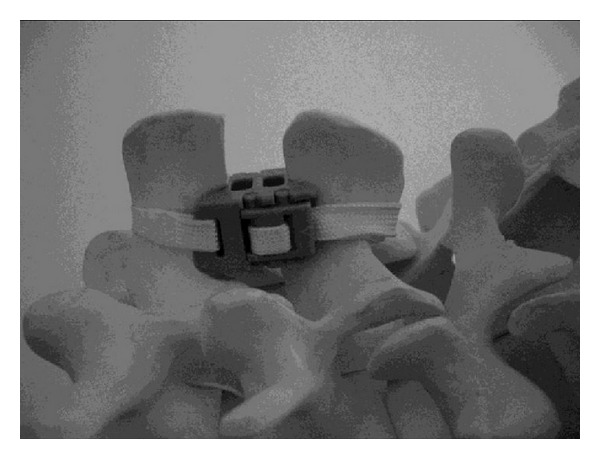
Model of a second generation Wallis implant with the polyester bands passed around the overlying and underlying interspinous ligaments and tightened.

*Wallis (Zimmer Spine, Formerly Abbot Spine, Inc., Austin, TX).* The device consists of an interspinous spacer made of polyetheretherketone (PEEK), which limits extension, and two woven dacron bands that secure the implant and limit flexion (Figure 4). The spacer has a distal and proximal 10 degrees inclined groove to better house the spinous processes and adapt to the anatomy of the spinous processes. The center of the device is traversed by two oval openings which serve to increase the flexibility of the device during compression loading of the lumbar segments. The flat polyester bands have an increased surface contact with the spinous processes, minimizing local concentration of contact stresses on the bone during flexion movements. The spacers have all the same width but the heights increases from 8, 10, 12, and 14 to 16. The Wallis device is indicated for the treatment of low back pain associated with degenerative disc diseases as well as lateral recess and central spinal stenosis.

Surgical technique used was as follows: surgery is performed with the patient under general anesthesia. Depending on the indication, the Wallis implant is placed either subsequent to a conventional posterior decompressive surgical procedure or in isolated fashion through a midline incision. The patient is placed in a prone neutral position of physiological lumbar lordosis. After skin incision, the supraspinous ligament is detached from the two spinous processes of the degenerative lumbar level with a scalpel and retracted intact with the underlying paravertebral muscles. If necessary, a decompressive procedure is performed. The interspinous ligament is removed with a gouge and, if bone trimming is necessary to improve seating of the implant, the inferior aspect of the upper spinous process at the junction with the lamina is trimmed to seat the spacer deeply as much as possible with the laminae.

After determining the size with a trial implant, the selected implant is placed in the interspinous space. With a sharp, curved instrument, the polyester bands are passed around the overlying and underlying interspinous ligaments, as close as possible to the instrumented spinous process. The bands are secured to the spacer and tightened: a small titanium ring is crimped onto each band to avoid fraying at the severed end. The supraspinous ligament is reinserted onto each spinous process with suture. 

*Viking (Sintea, Italy).* It is a dynamic interspinous system that allows for compression movements, lateral bending, and load transfer along the spine preserving the kinematic movements in the vertebral segment where it is implanted. It works at the same time as a shock adsorber. The device is made of PAEK, a biocompatible polymer, with stiffness similar to human cortical bone. It is anatomical shape consists of two concave shaped ends, and it is core is an elastic spring which can be deformed. The system is used to treat lumbar minor instabilities and to reduce the incidence of disc herniation recurrence after microdiscectomy of the affected level.

Surgical technique used was as follows: the patient is placed in prone position on a surgical frame avoiding hyperlordosis of the spinal segment to be operated on.

The inferior aspect of the spinous processes must be trimmed, if necessary, to facilitate insertion of the interspinous spacer. The bony junction between the spinous processes and the laminae may be trimmed to position the implant as anterior as possible to ensure a stable fit against the laminae.

**Figure 5:**
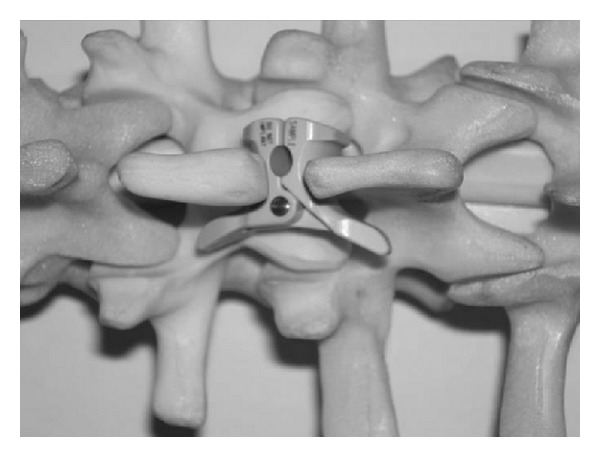
The BacJac interspinous device implanted in a lumbar spinal model.

Two bands are secured to the spacer and tightened: a small titanium ring is crimped onto each band to avoid fraying after cutting off the excess band (Figure 5).

**Figure 6:**
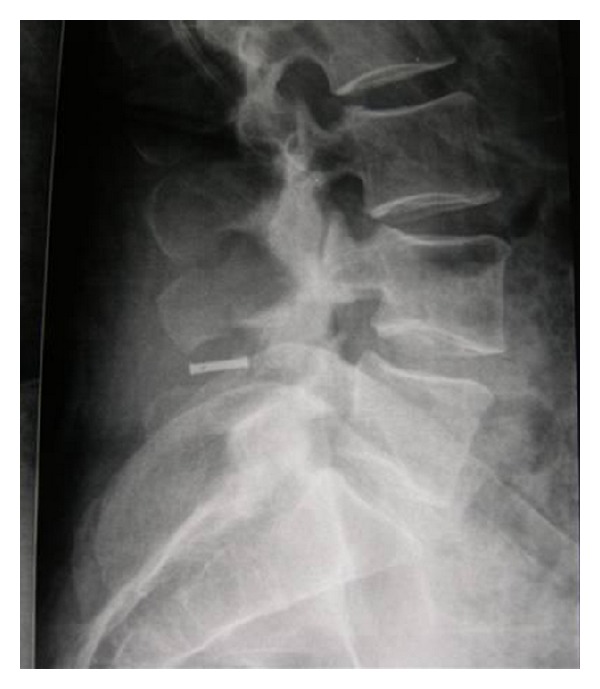
The lateral X-ray image reveals the radiographic marker of the BacJac implanted at L4-L5 interspinous area.

**Figure 7:**
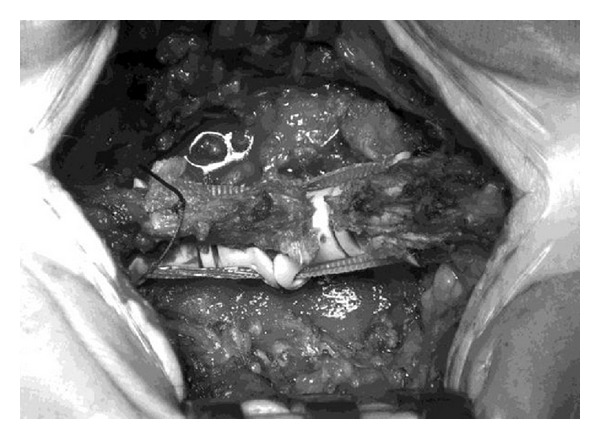
Intraoperative image of a double Viking implanted at higher lumbar levels. The bands are passed at the cranial and caudal interspinous level and tightened. When possible, the supraspinous ligament must be sutured.

*Ellipse (Sintea, Italy).* The Ellipse ISD is made of 2 elliptic components made of titanium and PAEK (plastic polymer) assembled by a click closure. The device has been developed to anatomically embrace both spinal processes in the PAEK surface in order to reduce the pull-out and respect the bone elasticity module. It must be applied between the spinous processes of the involved levels with monolateral MIS access (right or left, depending on the affected side) (Figures 6 and 7). This device is mainly used to expand the intervertebral space in mild and moderate lumbar stenosis. 

**Figure 8:**
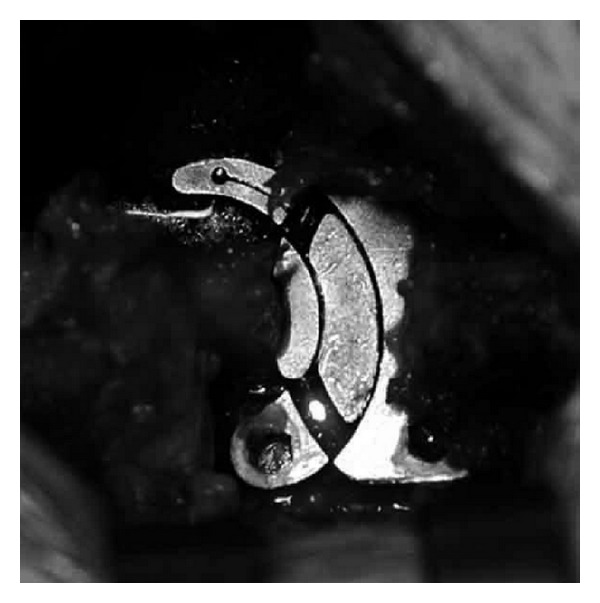
Intraoperative image of the first generation (prototype) all titanium Ellipse device. In this case the supraspinous ligament was removed to check the appropriate distraction of the interspinous area.

**Figure 9:**
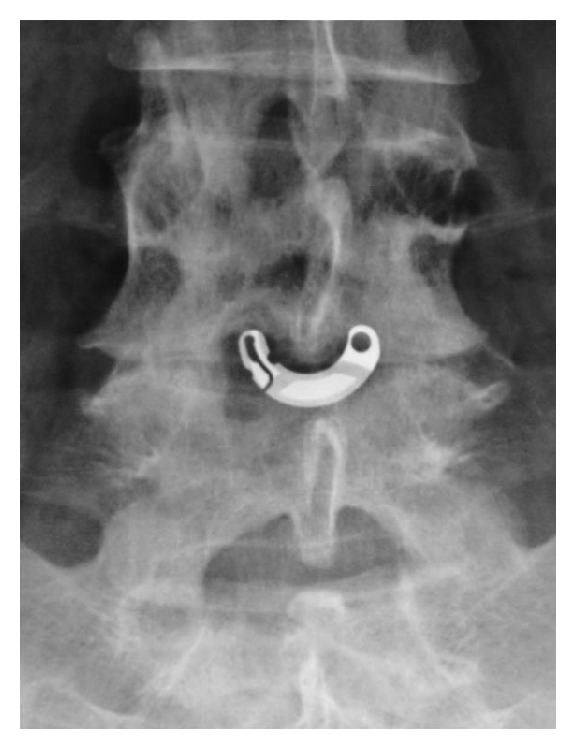
Anterior-posterior image of the second generation of Ellipse (half titanium/half PAEK) implanted at L4-L5.

*BacJac (Pioneer).* The BacJac is a minimally invasive device manufactured from PEEK, implanted through a unilateral surgical approach that reduces operating room and patient recovery time, while preserving future surgical options. The BacJac is a self-deploying, nonfusion device which is tissue sparing and ligament preserving. This device achieves spinal decompression by limiting the symptomatic extension while maintaining physiologic motion. Due to its large contact area with the spinous processes and its near-physiologic modulus, it ensures a minimal risk of subsidence (Figures 8 and 9). The best indication for this device seems to be radiculopathy and neurogenic claudication secondary to lumbar spine degenerated disc diseases.

Multiple companies have offered in recent years various devices, such as NuVasive (San Diego, CA) with ExtendSure and Biomech (Teipi, Taiwan) with the Promise and/or Rocker designs made of PEEK and mobile core and articulated design, respectively; Cousin Biotech (France) with the Biolig silicon encapsulated in woven synthetics device, Vertiflex (San Clemente, CA) with the Superion implant with deployable wings aiming at less invasive insertion that has started IDE study since 2008, Synthes (West Chester, PA) with the In-Space system with minimal insertion-that also started IDE in 2008 that was terminated at later date, or Orthofix (Bussolengo, Italy) with InSWing, Maxx Spine (Bad Schwalbach) with I-MAXX, Globus Medical (Audubon, PA) with Flexus, Privelop (Neunkirchen-Seelscheidm Germany) with Spinos.

### 4.2. Percutaneous Interspinous Devices

**Figure 10:**
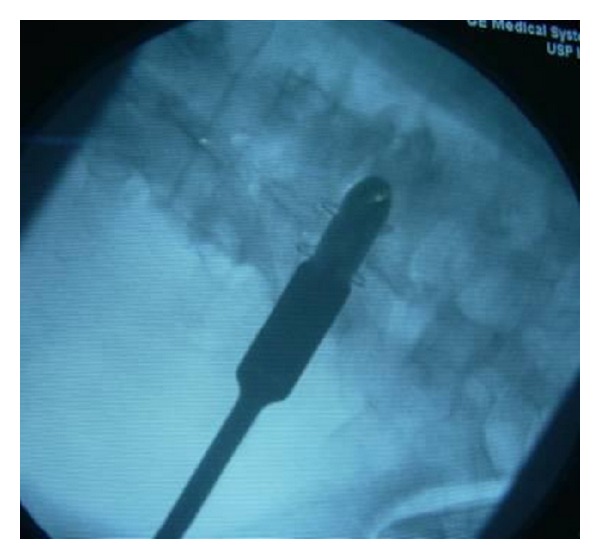
Anteroposterior radiographs of the lumbar spine during introperative radioscopy showing the insertion of an Aperius PercLID System at the L3-L4 level. The implant is preassembled on the inserter so it can be inserted without intermediate steps once the desired distraction is achieved. The implant core is manufactured of Titanium alloy (TiAl6V4 alloy) while the external shell is composed of pure Titanium.

**Figure 11:**
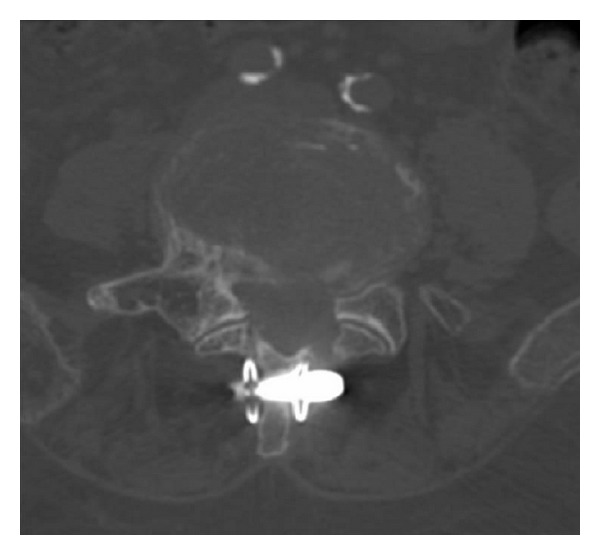
Axial CT image of the Aperius inserted at L4-L5 level showing the wings expanded on each side of the spinous process.

**Figure 12:**
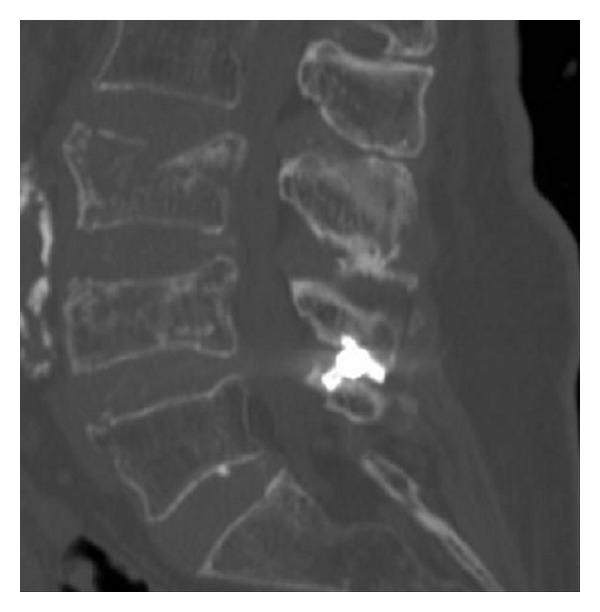
Sagittal CT image of the Aperius inserted at L4-L5 level.

**Figure 13:**
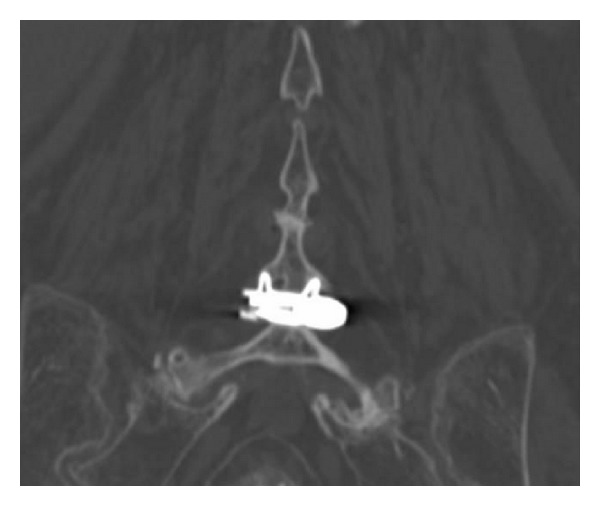
Coronal CT image of the Aperius inserted at L4-L5 level.

*Aperius (Medtronic).* The Aperius PercLID System is a percutaneous interspinous bullet-shaped implant. The system is composed of a set of color-coded distraction trocars of increasing sizes (8, 10, 12, and 14 mm) and of the preassembled inserter devices with implants. The 8-mm distraction trocar has a sharp pointed tip to facilitate piercing of the interspinous ligament for the subsequent trocars and for the implant. Each trocar and each inserter have a curved shape, which facilitates convenient access to the target level and positioning of the implant. Each implant is preassembled on the inserter so it can be inserted without intermediate steps once the desired distraction is achieved. The implant core is made of titanium (TiAl6V4) alloy, whereas the external shell is composed of commercially pure titanium. By turning the actuating handle of the inserter, a compressive force is created, retracting the outer shell and deploying the wings, which expand on each side of the spinous process, stabilizing the interspinous implant on the midline (Figures 10, 11, 12, and 13).

**Figure 14:**
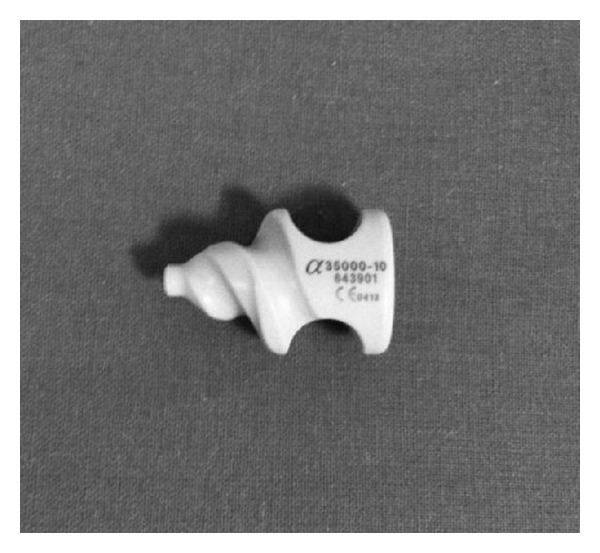
The self-distracting helical tip of the Helifix Interspinous Distraction System.

*Helifix (Alphatec Spine).* The Helifix Interspinous Spacer System is a percutaneous self-distracting implant manufactured from PEEK (polyetheretherketone) material and tantalum radiographic markers. It is composed of a self-distracting helical tip (Figure 14). Surgical technique is done through a posterior lateral approach, after a 2-3 cm incision; a guidewire is inserted under lateral fluoroscopy to find the interspinous space; a ligament splitter dilates through the interspinous ligament; then a dilator trial is positioned between the superior and inferior spinous processes. The insertion of increasing size trocars allows for a gradual distraction of the interspinous area to measure the optimal decompression and prevent overdistraction. Once proper fit is established, the Helifix implant is inserted with a rotating movement of the self-distracting helical tip in the interspinous area. This device stretches the ligamenta flava and the posterior fibers of the annulus fibrosus, thus enlarging the spinal canal in mild and moderate lumbar stenosis.

### 4.3. Interspinous Fusion Devices

Interspinous fusion devices contrast with interspinous distraction devices (also called spacers); the latter are used alone for decompression and may not be fixed to the spinous processes.

**Figure 15:**
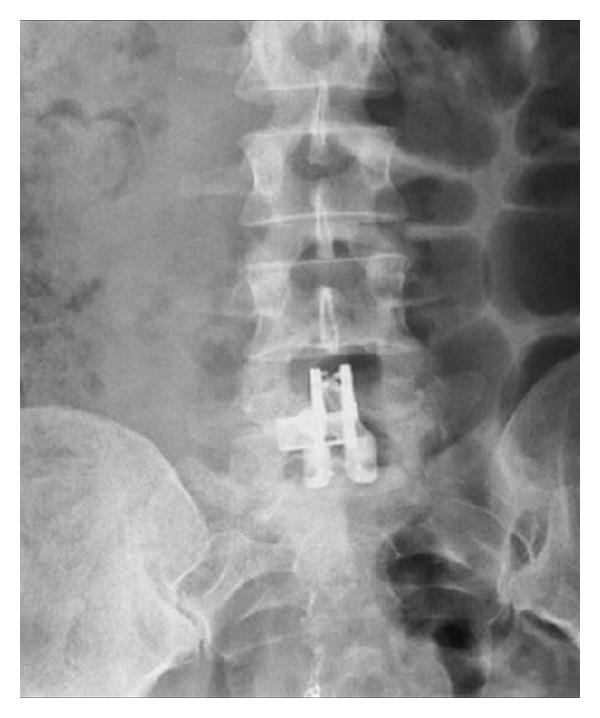
Postoperative anteroposterior X-ray image of Aspen shows the spikes in the lateral plates of the device for bone fixation.

*Aspen (Lanx).* The Aspen Device is an alternative to pedicle screws in achieving fusion; it delivers simplified posterior stabilization and renewed anatomical alignment through a minimally invasive implant and can be used in single- or multilevel constructs (Figure 15). Aspen is used alone or as an adjunct to interbody fusion and/or posterior fusion with decompression in treatment from T1-S1. It provides an alternative to more conventional means of fixation such as pedicle screws or anterior plates. This device is an alternative to dynamic interspinous spacers for the treatment of spinal stenosis and to conventional means of fixation to achieve fusion. Proprietary spiked-plate design provides reliable bone fixation. The interspinous implant serves to support the formation of fusion and decompression by fixation, load sharing, and interspinous process spacing, while decompressing spinal canal. It has an offset shape to accommodate multilevel placement with a wide range of sizes for patient variations. 

**Figure 16:**
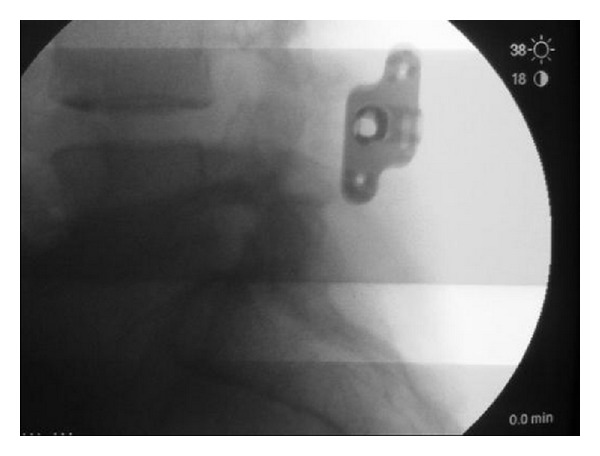
Intraoperative X-ray image of a BacFuse implanted at L4-L5 interspinous level.

*BacFuse (Pioneer).* This device is used alone or as an adjunct to interbody fusion and/or posterior fusion with decompression in treatment from T1-S1. It has a spiked-plate design that provide spinous process fixation. The BacFuse decompresses the spinal canal while supporting the formation of interspinous fusion (Figure 16). 

**Figure 17:**
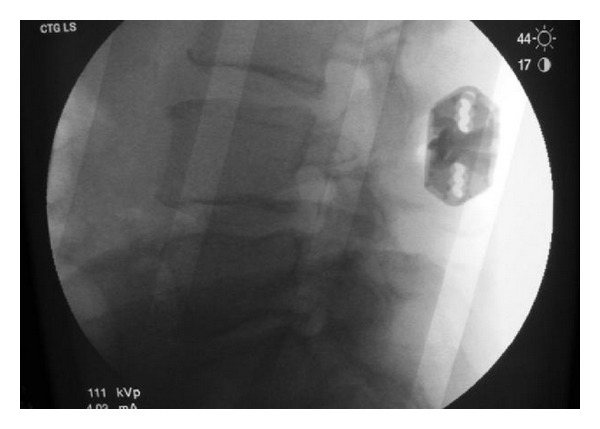
Intraoperative X-ray image of a Stabilink implanted at L3-L4 interspinous level.

*Stabilink (Southern Spine).* The implant has a small diameter wide-spike design with 16 spikes per implant over a broad area. There are three different implant designs and a wide range of sizes for an optimum anatomical fit. The anterior design maximizes containment area for bone graft material to optimize bony fixation (Figure 17).

#### 4.3.1. Expandable Interspinous Fusion Devices

**Figure 18:**
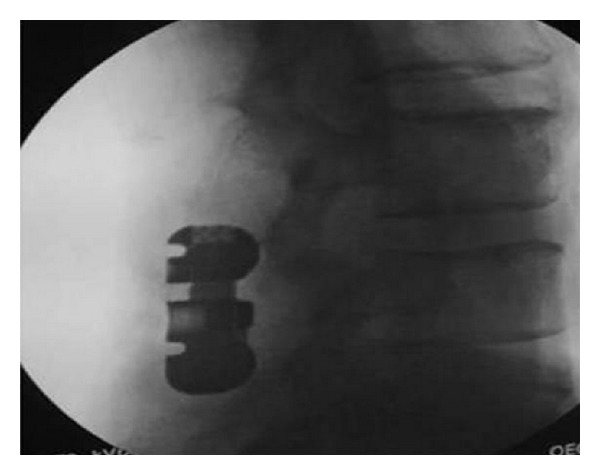
Intraoperative lateral X-ray of the BridgePoint System with the telescoping plates that fixate and compress spinous processes restoring sagittal alignment.

*BridgePoint (Alphatec Spine).* The BridgePoint is an advanced spinous process fixation system that was developed to address some of the disadvantages of traditional stabilization devices. The implant has unique telescoping plates that allow surgeons to fixate and compress spinous processes to restore sagittal alignment and facilitate a reliable interbody fusion (Figure 18). The device's large contact area provides a strong anchor point from which is possible to apply compression between the adjacent spinous processes during the surgical procedure and it offers optimal stability during the fusion process. The system is easy to use and the quick procedure offers minimal exposure, dissection, muscle trauma, and blood loss as well as protection of neural structures. The device has a large bone graft window and is intended for use with bone graft material and is not intended for stand-alone use. Surgical technique was as follows: after a midline exposure of the spinous processes, the supraspinous and interspinous ligaments are removed entirely. Bilateral hemilaminectomies and partial medial facetectomies are done preserving the laminae, facets, and spinous processes. The facing surfaces of the spinous processes are decorticated. The interspinous space distance is determined using a measuring guide. The appropriate sized interspinous device is chosen and placed between the spinous processes and squeezed together, and the device is compressed or distract with an appropriate device. The space between the spinous processes and within the space between the BridgePoint plates is filled with allograft bone product. 

**Figure 19:**
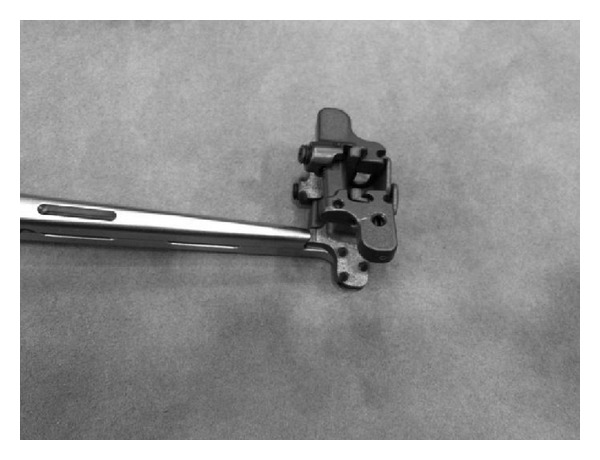
The Posterior Fusion System is a titanium implant that has an adjustable, fenestrated core and adjustable-length plates which allows for expansion and compression.

*Posterior Fusion System (Lanx).* The posterior Fusion device consists of spinous process plates made of Titanium Alloy and commercially pure titanium (Figure 19). It is intended to provide stabilization in the lumbar and thoracic spine as an adjunct to interbody and/or posterior fusion, or as stand-alone device. The device is designed to support the formation of fusion and decompression by fixation and interspinous process spacing, while renewing anatomic alignment. The implant has an adjustable, fenestrated core and adjustable-length plates which allow for expansion and compression. The in situ compressibility allows surgeons to control the lordosis at the treated level, while the adjustable sizing allows for an optimized anatomical fit. Bone graft material is then packed within the hollow post of the implant.

## 5. Pathologies Treated

Interspinous fixation systems are less invasive and present fewer risks than pedicle or facet screws in combination with fusion for the treatment of degenerative lumbar diseases. Biomechanical studies suggest that interspinous fusion implants may be similar to pedicle screw-rod constructs in limiting the range of flexion-extension, but they may be less effective than bilateral pedicle screw-rod fixation for limiting axial rotation and lateral bending.

## 6. IPD Compared with Other Treatments

### 6.1. IPD Compared with Nonoperative Treatment

FDA approval of X-Stop was based on a multicenter, prospective randomized controlled clinical study: patients were randomized to X-Stop (*n* = 100) or to a control group (*n* = 91) which received continued nonoperative therapy, including bed rest, a lumbar corset, and epidural injections [38]. At two years, the Symptom Severity score for the X-Stop and the control group was 45.4% above baseline scores and 7.4%, respectively; the mean physical function score changes were 44.3% and −0.4%, respectively. In another report, published by the same authors, the X-Stop group showed improvements in physical and mental component scores (Quality of life SF-36) compared to both baseline and control patients. But in this paper, the beneficial outcomes reported were misleading inflated and, in addition, there was a conflict of interest for the two primary authors. Anderson reported two years outcome in patients whose symptoms were due to degenerative spondylolisthesis at one or two levels; using ZCQ and SF-36 questionnaire, 63.4% of patients in the X-Stop group met success criteria while 12.9% of the control group were satisfied. In all these studies, while the short-term results are encouraging, it is not possible to reach scientific conclusions related to long-term health outcomes.

### 6.2. IPD Compared with Decompressive Surgery

The IDE (investigational device exemption) trial for the Coflex was a randomized multicenter noninferiority study that compared Coflex implantation with decompression and posterolateral fusion with pedicle screw fixation [64]. Patients were randomized in a 2 : 1 ratio: noninferiority between Coflex and pedicle screws was reported, with 66.2% success with Coflex and 57.7% success with fusion. ZCQ success was achieved in 78.3% of Coflex patients compared with 67.4% of control group. The percentage of adverse events was 5.6% for both groups with a reoperation rate of 10.7% in the Coflex group and 7.5% in the fusion group. In another randomized trial of 100 cases with lumbar stenosis, patients were randomized in a 1 : 1 ratio to undergo either X-Stop implantation or surgical decompression. Although at 24 months follow-up there was no significant difference in scores for symptoms and function, reoperation rates were higher in the X-Stop group (26%) than in the decompression group (6%) [65].

### 6.3. IPD versus IPD

Wilke compared four different interspinous implants (Wallis, Diam, Coflex, and X-Stop) in terms of their flexibility and intradiscal pressure [8]. They found that they all had similar effect on the flexibility, reducing the intradiscal pressure in extension, but having no effect in flexion, lateral bending, and axial rotation. Sobottke et al. compared retrospectively the clinical and radiological results of three different IPD (Diam, X-Stop, and Wallis) [31]. The foraminal height, foraminal width, and foraminal cross-sectional area were significantly increased after surgery with all the three devices, but progressively decreased during follow-up. They reported that the X-Stop group showed a significantly larger foraminal cross-sectional area and height than the other two devices. The best pain relief, but not statistically significant, was noted for patients who received the Diam, followed by the X-Stop and the Wallis devices.

## 7. Cost-Effectiveness of ISP

First study published examining the cost of laminectomy versus ISP surgery was in 2007 [40]. Kondrashov reported that X-Stop was significantly more cost-effective than laminectomy. But in their study, they used the cost perspective of the hospital rather than that of the society; in addition, the senior author of the study was one of the inventors of the X-Stop device and had financial ties to the manufacturer. It has been reported recently [66] in a health economical analysis that considerable healthcare cost savings can be obtained using an IPD on an outpatient basis. Following standard cost-effectiveness principles, Burnett published a literature review comparing the costs of conservative treatment, decompressive laminectomy, and X-Stop implantation in patients with lumbar spinal stenosis. They suggested that laminectomy was the most effective treatment strategy, followed by X-Stop and then conservative treatment at a 2-year time horizon [67]. For single level surgery, laminectomy was more effective, but X-Stop was less costly. The cost difference was secondary to the fact that laminectomy was performed as an inpatient surgery, whereas X-Stop was performed as an ambulatory setting. But for double-level procedure, laminectomy was less costly and more effective than X-Stop. Relative effectiveness and cost treatment strategies for lumbar stenosis revealed for 1-level procedure a mean cost of $9.291 for laminectomy, $7.900 for X-Stop, and $3.478 for conservative treatment; for 2-level procedures, the mean cost was $9.329 for laminectomy, 13.429 for X-Stop, and $3.435 for conservative treatment. In Epstein's series, the average charge for X-Stop devices ranged from $17.600 for one-level procedures to $57.201 for three-levels procedures; additionally, the average operating room charge/patient was $3908 (average time 2.1 hours) and the average recovery room charge was $1151/patient (average 4.6 hours) [68].

## 8. Complications

Interspinous process spacers have been introduced as a possible alternative to spinal decompression and fusion for the treatment of lumbar spinal stenosis and discogenic lower back pain. Although lumbar canal decompression with laminectomy and fusion have shown to offer a good outcome, it is a rather invasive procedure and some long-term clinical studies report a high rate of complications. In 1992, an extensive meta-analysis of the literature of spinal stenosis surgery reported by Turner et al. showed the following complication rate for lumbar decompressive surgery: dural tears 5,9%, superficial infection 2,3%, deep infection 1,1%, perioperative mortality 0,3%, and deep vein thrombosis 2,7% for an overall complication rate of 12,6% [78]. The overall complication rate in X-Stop surgery amounts in some series for 3,3%, including fracture of the spinous processes, dislocation of the prosthesis, and skin infections whereas such rate is 9,7% for decompressive laminectomies [79]. More recently, Bowers et al. showed a long-term complication rate of 38%, with 11 (85%) of 13 patients requiring additional spine surgery after X-Stop placement [80]. They observed a higher rate of spinous process fracture (23%) than previously reported.

In a retrospective study done by Tuschel et al., a fairly high revision rate (30.4%) was observed [81]. Verhoof et al. described the outcome of X-Stop placement in a group of patients with Grade I spondylolisthesis, documenting a high rate (58%) of failure [71] (Table 3). We found an increasing number of recent studies suggesting that IPD may not be as free of complications and reoperations as previously reported in the first studies. Three main causes of failure are reported in the literature: errors of indications, technical errors, and structural failure of the implant.

## 9. Conclusions 

The increasing use of interspinous implants, combined with a growing older population, has raised questions from the scientific community. While the rationale of their use in the treatment of spinal stenosis is clear, the role in the treatment of degenerative disc disease remains to be defined. One proposed mechanism of action is unloading of the posterior annulus by distraction. Interspinous devices with shock absorption and postoperative adjustability may present the future of these devices. However, coupling surgical decompression techniques with the use of interspinous devices has added confusion to the contribution of interspinous devices in pain/symptom relief. The 2011 clinical guidelines from the NASS (North American Spine Society) suggested that there is insufficient evidence at this time to make a recommendation for or against the placement of an interspinous process spacing device in patients with lumbar spinal stenosis. The American pain Society guidelines indicated that interspinous spacer device have a B recommendation: the net benefit is considered moderate through two years, with insufficient evidence to estimate the net benefit for long-term outcomes [82, 83]. Current evidence is not sufficient to permit conclusions whether any beneficial effect from interspinous process decompression provides significant advantages over laminectomy, which is the current standard of care for surgical decompression of lumbar spinal stenosis. Interspinous process decompression is still considered investigational and poor clinical results in the medical literature will continue to limit the appeal of these devices to many surgeons in the future. But, because of the low invasiveness of the surgical implantation of the interspinous devices, this technique seems to have robust pathophysiological grounding and promises to play an important role in the future degenerative lumbar microsurgery, especially in the older population.

## Figures and Tables

**Table:** (a)

	X-Stop	Coflex	DIAM	Wallis	BacJac	Viking	Ellipse	Aperius
Producer	Medtronic	Paradigm Spine	Medtronic	Zimmer	Pioneer	Sintea	Sintea	Medtronic
Category	Static	Dynamic	Dynamic	Static	Static	Dynamic	Static	Static
Material	Titanium	Titanium	Silicon	Peek	Peek	Paek	Titanium/Paek	Titanium
Approach	Bilateral	Bilateral	Monolateral	Bilateral	Monolateral	Bilateral	Monolateral	Percutaneous
Fixation	Wings	Wings	Ribbons	Ribbons	Clip	Ribbons	Clip	Wings
Preservation supraspinous ligament	Yes	No	Yes	No	Yes	No	Yes	Yes

**Table:** (b)

Helifix	Aspen	BacFuse	Stabilink	Bridge Point	Posterior Fusion System
Alphatec Spine	Lanx	Pioneer	Southern Spine	Alphatec Spine	Lanx
Static	Static/Fusion	Static/Fusion	Static/Fusion	Expandable/Fusion	Expandable/Fusion
Peek	Titanium	Titanium	Titanium	Titanium	Titanium
Percutaneous	Bilateral	Bilateral	Bilateral	Bilateral	Bilateral
Helical tip	Spikes	Spikes	Spikes	Spikes	Spikes
Yes	Yes	Yes	Yes	Yes	Yes

**Table 2:** Relevant Biomechanical studies on interspinous devices.

Author	Device	Year	Cases	Study	Evaluation	Results
Swanson et al. [52]	X-Stop	2003	8	Cadaver	Intradiscal pressure	Unloading of the disc

Lee et al. [35]	X-Stop	2004	10	Case series	Foraminal and spinal canal area	Increase of 22.3% spinal canal and 36.5% foraminal area

Zucherman et al. [38]	X-Stop	2005	191	Case series	X-ray assessment	No differences between 12 and 24 months

Richards et al. [37]	X-Stop	2005	8	Cadaver	Foraminal and spinal canal area	Increase of 18% spinal canal and 25% foraminal area in extension

Fuchs et al. [55]	X-Stop	2005	7	Cadaver	Assessment after facetectomy	Bilateral facetectomy increases range of motion

Wiseman et al. [53]	X-Stop	2005	7	Cadaver	Facet loading	Reduced mean peak pressure

Siddiqui et al. [45]	X-Stop	2006	26	Case series	Kinematics	Significant increase of spinal canal and foraminal area

Phillips et al. [60]	Diam	2006	6	Cadaver	Kinematics after facetectomy and discectomy	Reduces increased segmental motion after discectomy

Tsai et al. [42]	Coflex	2006	8	Cadaver	Kinematics	Returns a partially destabilized spine back to intact condition

Kong et al. [61]	Coflex	2007	42	Case series	Adjacent segment motion compared with PLIF	Increased in PLIF

Kim et al. [62]	Diam	2007	62	Case series	Disc height	No changes in disc height at 12 months

Lafage et al. [63]	Wallis	2007	6	Cadaver	Kinematics	Lower stress in disc fibers

Wilke et al. [8]	X-Stop/Diam	2008	24	Cadaver	Intradiscal pressure	Reduction of intradiscal pressure in extension

**Table 3:** Relevant clinical studies on interspinous devices.

Author	Device	Year	Cases	Study	Follow up	Results
Zucherman et al. [36]	X-Stop	2004	191	Prospective comparative	12 months	Good outcome (implant group 59%/control 12%)

Lee et al. [35]	X-Stop	2004	10	Prospective noncomparative	11 months	Satisfaction 70%

Zucherman et al. [38]	X-Stop	2005	191	Prospective comparative	24 months	Good outcome in implant group

Anderson et al. [69]	X-Stop	2006	75	Prospective comparative	24 months	Good outcome (implant group 63%/control 13%)

Kondrashov et al. [40]	X-Stop	2006	18	Prospective noncomparative	51 months	Good outcome 78%

Hsu et al. [39]	X-Stop	2006	191	Prospective comparative	24 months	Better outcome in implant group

Taylor et al. [70]	Diam	2007	104	Retrospective noncomparative	18 months	Analgesic reduced in 63.1%

Kim et al. [62]	Diam	2007	62	Retrospective noncomparative	12 months	Improvement in pain scores

Siddiqui et al. [45]	X-Stop	2007	40	Prospective noncomparative	12 months	Reoperation 8%; satisfied 71%

Kong et al. [61]	Coflex	2007	42	Retrospective-prospective comparative	12 months	Clinical improvement in both groups

Verhoof et al. [71]	X-Stop	2008	12	Retrospective noncomparative	30 months	Reoperation 58%

Sénégas et al. [72]	Wallis	2009	107	Retrospective noncomparative	13 years	Good outcome 80%; reoperation 20%

Kuchta et al. [73]	X-Stop	2009	175	Prospective noncomparative	24 months	VAS and ODI decreased 39% and 20.3%

Sobottke et al. [31]	X-Stop	2009	129	Retrospective noncomparative	10 months	Good symptom control

Barbagallo et al. [74]	X-Stop	2009	69	Retrospective noncomparative	23 months	Reoperation of 7 cases

Richter et al. [75]	Coflex	2010	60	Prospective comparative	12 months	No differences

Galarza et al. [30]	Aperius	2010	40	Prospective noncomparative	12 months	Satisfaction 90%

van Meirhaeghe et al. [76]	Aperius	2013	156	Prospective noncomparative	12 months	High clinical improvement in 58%; reoperation 9%

Surace et al. [77]	Aperius	2013	37	Retrospective noncomparative	18 months	VAS and ODI decreased 5% and 26%; reoperation 5.6%
